# Supplementary material for: Bacterial microcompartment utilisation in the human commensal Escherichia coli Nissle 1917
Source: J Bacteriol. Author manuscript; Available in PMC 2024 Dec 17. (PMC7617246; doi:10.1128/jb.00269-24)
Supplement: Supplementary Information [file EMS201728-supplement-Supplementary_Information.pdf]

# Supplementary Information: Bacterial microcompartment utilisation in the human commensal *Escherichia coli* Nissle 1917

Chania Clare[1], Jack W. Rutter[1], Alex J.H. Fedorec[1], Stefanie Frank[2], and Chris P. Barnes[1,3]

[1] *Department of Cell and Developmental Biology, University College London, London, UK*
[2] *Department of Biochemical Engineering, University College London, London, UK*
[3] *Department of Genetics, Evolution and Environment, University College London, London, UK*

E-mail: christopher.barnes@ucl.ac.uk

## 1 Extended materials and methods

**Tables S1-S3** contain statistical analysis, **Table S4** contains *eut* operon protein sequence homology between EcN and EcBW. **Tables S5-S7** contain summaries of models used in Flux Balance Analysis.

Table S 1: **Test statistics corresponding to values from Fig 2F, where the carry capacity is estimated with the Gompertz function ± standard error, where each condition for each species if compared to EA + $B_{12}$**

| Species | Condition | Estimated Carry Capacity | P-value |
|---|---|---|---|
| EcBW | No addition | 0.119 ± 0.00100 | 0.54 |
| | EA | 0.123 ± 0.00184 | 0.66 |
| | $B_{12}$ | 0.130 ± 0.00128 | 0.08 |
| | EA + $B_{12}$ | 0.121 ± 0.00356 | |
| EcN | No addition | 0.140 ± 0.000980 | 0.0000055 |
| | EA | 0.126 ± 0.00199 | 0.0000053 |
| | $B_{12}$ | 0.120 ± 0.00118 | 0.0000033 |
| | EA + $B_{12}$ | 0.262 ± 0.00367 | |
| Sent | No addition | 0.0793 ± 0.000516 | 0.00000029 |
| | EA | 0.0811 ± 0.000413 | 0.00000029 |
| | $B_{12}$ | 0.0795 ± 0.000526 | 0.00000029 |
| | EA + $B_{12}$ | 0.341 ± 0.00384 | |

Table S 2: **Test statistics corresponding to values from Fig 3B-C, where the mean fold change of cultures supplemented with EA + $B_{12}$ are compared to those supplemented with glucose instead, ± SEM.**

| Species | Gene | Mean Fold Change | p-value |
|---|---|---|---|
| EcN | EutS | 30.051 ± 0.877 | 0.0000085 |
| | EutB | 25.748 ± 0.890 | 0.000011 |
| | EutR | 12.740 ± 0.879 | 0.000000073 |
| EcBW | EutS | 219.176 ± 0.803 | 0.00000011 |
| | EutB | 1.245 ± 0.812 | 0.75 |
| | EutR | 170.989 ± 0.822 | 0.0000015 |

Table S 3: **Test statistics corresponding to values from Fig 4E, where the mean $NH_4^+$ concentration ± SEM is compared within each species when grown in media supplemented with either EA + $B_{12}$ or glucose as a carbon source.**

| Species | Condition | Mean [$NH_4^+$] | p-value |
|---|---|---|---|
| EcN | EA + $B_{12}$ | 0.0360 ± 0.000649 | 0.000088 |
| | Glucose | 0.0228 ± 0.000505 | |
| Sent | EA + $B_{12}$ | 0.0222 ± 0.000841 | 0.5353 |
| | Glucose | 0.0216 ± 0.000278 | |

Table S 4: **Protein sequence homology of proteins comprising *eut* operon between EcN and BW25113**

| Protein | No. amino acids | % identity between EcN and BW25113 |
|---|---|---|
| eutS | 111 | 100.00 |
| eutP | 159 | 97.48 |
| eutQ | 233 | 97.00 |
| eutT | 267 | 100.00 |
| eutD | 338 | 99.70 |
| eutM | 97 | 100.00 |
| eutN | 95 | 100.00 |
| eutE | 467 | 98.93 |
| eutJ | 278 | 98.56 |
| eutG | 395 | 98.73 |
| eutH | 408 | 99.02 |
| eutA | 467 | 98.07 |
| eutB | 453 | 99.78 |
| eutC | 295 | 99.66 |
| eutL | 219 | 97.26 |
| eutK | 166 | 98.80 |
| eutR | 350 | 98.86 |

Table S 5: **Summary of each model used in this study**

| | **iJO1366** BW25113 2011 | **iHM1533** EcN 2022 | **STM_v1.0** Sent 2011 |
|---|---|---|---|
| Chromosome size of strain (Mb) | 4.63 | 5.44 | 4.86 |
| Number of reactions | 2583 | 3143 | 2546 |
| Number of metabolites | 1805 | 2261 | 1802 |
| Number of genes | 1367 | 1533 | 1271 |
| Compartments | cytosol; extracellular space; periplasm | cytosol; extracellular space; periplasm | cytosol; extracellular space; periplasm |

Table S 6: **Inclusion of metabolites of the *eut* BMC metabolic pathway within each model.** Metabolite ID and names are given as standard GEM notation, and each model name is given alongside its species and year the model was created.

| Metabolite Name | Metabolite ID | iJO1366 EcBW 2011 | iJO1366_CC EcBW 2024 | iHM1533 EcN 2022 | iHM1533_CC EcN 2024 | STM v1.0 Sent 2011 |
|---|---|---|---|---|---|---|
| Acetaldehyde | acald_e | × | × | × | × | × |
| Acetaldehyde | acald_c | ✓ | ✓ | ✓ | ✓ | ✓ |
| Acetyl phosphate | actp_c | ✓ | ✓ | ✓ | ✓ | ✓ |
| Acetyl-CoA | accoa_c | ✓ | ✓ | ✓ | ✓ | ✓ |
| Ammonium | nh4_e | ✓ | ✓ | ✓ | ✓ | ✓ |
| Ammonium | nh4_p | ✓ | ✓ | ✓ | ✓ | ✓ |
| Ammonium | nh4_c | ✓ | ✓ | ✓ | ✓ | ✓ |
| Coenzyme A | coa_c | ✓ | ✓ | ✓ | ✓ | ✓ |
| Ethanol | etoh_e | ✓ | ✓ | ✓ | ✓ | ✓ |
| Ethanol | etoh_p | ✓ | ✓ | ✓ | ✓ | ✓ |
| Ethanol | etoh_c | ✓ | ✓ | ✓ | ✓ | ✓ |
| Ethanolamine | etha_e | ✓ | ✓ | ✓ | ✓ | ✓ |
| Ethanolamine | etha_p | ✓ | ✓ | ✓ | ✓ | ✓ |
| Ethanolamine | etha_c | ✓ | ✓ | ✓ | ✓ | ✓ |
| H+ | h_e | ✓ | ✓ | ✓ | ✓ | ✓ |
| H+ | h_p | ✓ | ✓ | ✓ | ✓ | ✓ |
| H+ | h_c | ✓ | ✓ | ✓ | ✓ | ✓ |
| NAD | nad_c | ✓ | ✓ | ✓ | ✓ | ✓ |
| NADH | nadh_c | ✓ | ✓ | ✓ | ✓ | ✓ |
| Phosphate | pi_e | ✓ | ✓ | ✓ | ✓ | ✓ |
| Phosphate | pi_p | ✓ | ✓ | ✓ | ✓ | ✓ |
| Phosphate | pi_c | ✓ | ✓ | ✓ | ✓ | ✓ |

Table S 7: **Inclusion of reactions of the *eut* BMC metabolic pathway within each model.** Reaction ID and names are given as standard GEM notation, and each model name is given alongside its species and year the model was created.

| Reaction ID | Reaction | iJO1366 EcBW 2011 | iJO1366_CC EcBW 2024 | iHM1533 EcN 2022 | iHM1533_CC EcN 2024 | STM v1.0 Sent 2011 |
|---|---|---|---|---|---|---|
| Ex_acald_e | acald_e <=> | ✓ | ✓ | ✓ | ✓ | ✓ |
| ALCD2x | etoh_c + nad_c <=>acald_c + h_c + nadh_c | ✓ | ✓ | ✓ | ✓ | ✓ |
| EX_nh4_e | nh4_e <=> | ✓ | ✓ | ✓ | ✓ | ✓ |
| NH4tpp | nh4_p <=>nh4_c | ✓ | ✓ | ✓ | ✓ | ✓ |
| NH4tex | nh4_e <=>nh4_p | ✓ | ✓ | ✓ | ✓ | ✓ |
| Ex_etoh_e | etoh_e <=> | ✓ | ✓ | ✓ | ✓ | ✓ |
| ETOHtrpp | etoh_p <=>etoh_c | ✓ | ✓ | ✓ | ✓ | ✓ |
| ETOHtex | etoh_e <=>etoh_p | ✓ | ✓ | ✓ | ✓ | ✓ |
| ETHAAL | etha_c –>acald_c + nh4_c | ✓ | ✓ | ✓ | ✓ | ✓ |
| EX_etha_e | etha_e –> | ✓ | ✓ | ✓ | ✓ | ✓ |
| ETHAt2pp | etha_p + h_p –>etha_c + h_c | ✓ | ✓ | ✓ | ✓ | ✓ |
| ETHAtex | etha_e <=>etha_p | ✓ | × | × | ✓ | ✓ |
| Ex_h_e | h_e <=> | ✓ | ✓ | ✓ | ✓ | ✓ |
| Ex_pi_e | pi_e <=> | ✓ | ✓ | ✓ | ✓ | ✓ |
| PItex | pi_e <=>pi_p | ✓ | ✓ | ✓ | ✓ | ✓ |
| PTAr | accoa_c + pi_c <=>actp_c + coa_c | ✓ | ✓ | ✓ | ✓ | ✓ |
| Htex | h_e <=>h_p | ✓ | ✓ | ✓ | ✓ | ✓ |

# 2 Extended results

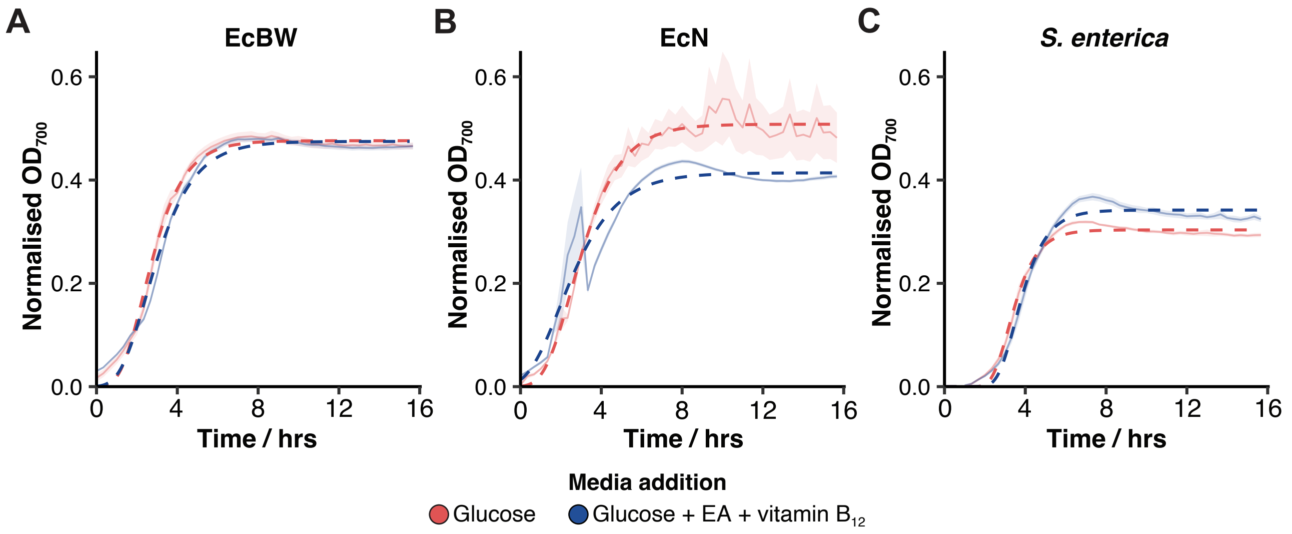


Figure S 1: **EA-dependent growth in *E. coli* Nissle 1917.** Growth curves of EcBW (**A**), EcN (**B**), and *S. enterica* (**C**) when grown in M9 supplemented with either glucose alone, or EA, vitamin $B_{12}$ and glucose, in triplicate. The mean and standard error are shown with a solid line and a pale ribbon, respectively, and the data was fitted with the Gompertz function shown by the respective dashed lines.

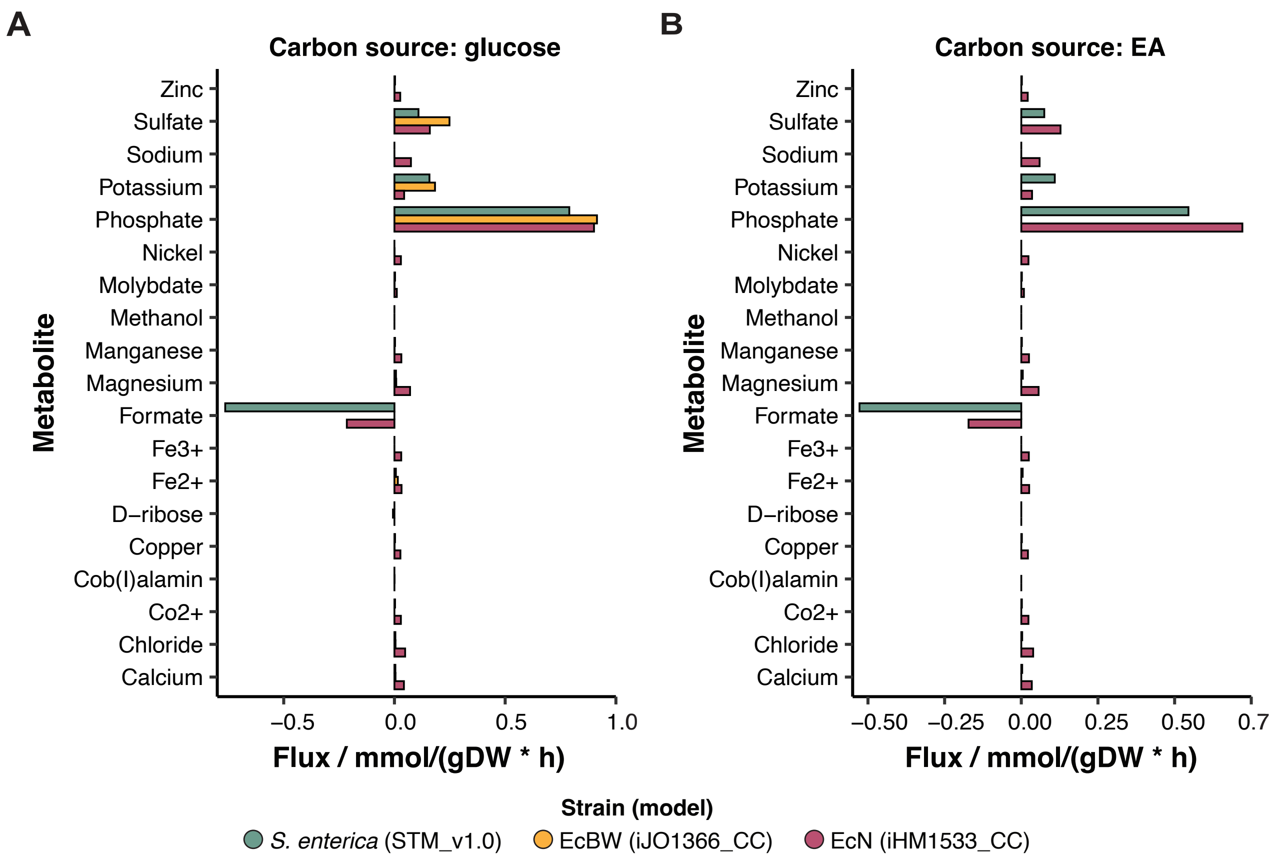


Figure S 2: **Metabolite flux prediction in *E. coli* Nissle 1917.** (**A & B**) Predicted flux from the FBA when the model is supplied with either EA or glucose as the sole carbon source, respectively .
